# Juxtapose: a gene-embedding approach for comparing co-expression networks

**DOI:** 10.1186/s12859-021-04055-1

**Published:** 2021-03-16

**Authors:** Katie Ovens, Farhad Maleki, B. Frank Eames, Ian McQuillan

**Affiliations:** 1 Department of Computer Science, University of Saskatchewan, Saskatoon, S7N 5C9 Canada; 2 Augmented Intelligence & Precision Health Laboratory (AIPHL), Research Institute of the McGill University Health Centre, Montreal, H4A 3S5 Canada; 3 Department of Anatomy, Physiology, and Pharmacology, University of Saskatchewan, Saskatoon, S7N 5E5 Canada

**Keywords:** Gene co-expression networks, Transcriptomics, Evolution, Machine learning, Embedding, Word2vec

## Abstract

**Background:**

Gene co-expression networks (GCNs) are not easily comparable due to their complex structure. In this paper, we propose a tool, Juxtapose, together with similarity measures that can be utilized for comparative transcriptomics between a set of organisms. While we focus on its application to comparing co-expression networks across species in evolutionary studies, Juxtapose is also generalizable to co-expression network comparisons across tissues or conditions within the same species.

**Methods:**

A word embedding strategy commonly used in natural language processing was utilized in order to generate gene embeddings based on walks made throughout the GCNs. Juxtapose was evaluated based on its ability to embed the nodes of synthetic structures in the networks consistently while also generating biologically informative results. Evaluation of the techniques proposed in this research utilized RNA-seq datasets from GTEx, a multi-species experiment of prefrontal cortex samples from the Gene Expression Omnibus, as well as synthesized datasets. Biological evaluation was performed using gene set enrichment analysis and known gene relationships in literature.

**Results:**

We show that Juxtapose is capable of globally aligning synthesized networks as well as identifying areas that are conserved in real gene co-expression networks without reliance on external biological information. Furthermore, output from a matching algorithm that uses cosine distance between GCN embeddings is shown to be an informative measure of similarity that reflects the amount of topological similarity between networks.

**Conclusions:**

Juxtapose can be used to align GCNs without relying on known biological similarities and enables post-hoc analyses using biological parameters, such as orthology of genes, or conserved or variable pathways.

**Availability:**

A development version of the software used in this paper is available at https://github.com/klovens/juxtapose

**Supplementary Information:**

The online version contains supplementary material available at 10.1186/s12859-021-04055-1.

## Background

High-throughput techniques such as RNA-seq and microarray make it possible to measure the expression level of a large number of genes in a single experiment. These high-throughput expression studies have resulted in a large number of gene expression datasets that are available through public repositories such as GEO [1] and ArrayExpress [2]. Differential expression analysis, which refers to the comparison of the expression measures of individual genes across phenotypes/conditions, has been the common practice in analysing these data [3]. This approach only leads to the identification of individual genes with different expression levels across phenotypes/conditions. However, often coordinated interaction of groups of genes drives various biological processes and functions, and the change in the expression level of a single gene does not capture this complex network of interactions. These complex gene-gene interactions can be modeled as a network.

Networks have been widely used for the study of complex interactions between genes, proteins, and other biomolecules [4, 5]. In particular, gene co-expression networks (GCN) constructed using gene expression data can be utilized to extract information about coordinately expressed genes. GCNs are commonly represented as weighted networks, where the networks have a numeric value representing each interaction among nodes (genes) by some measure of their relationship as opposed to being represented as a binary network—typically used to represent protein-protein interaction (PPI) networks, for example—with unweighted links among nodes of a network. It has been shown that co-expression networks are not static, and can change depending on the the biological context [6]. Comparing these networks can aid in improving functional annotation of genes and the discovery of gene–gene interactions [7], revealing the molecular mechanisms of complex diseases or the relationships between biological processes [8], and helping to speed up the process of selecting genes for targeted mutational studies [6]. Therefore, comparing these networks can provide valuable insight into the key coordinated interactions that are associated with the phenotypes under study.

Approaches capable of comparing dense and weighted networks such as GCNs include measuring the similarity between the topological properties of networks [9–12], clustering for the identification of conserved modules of genes [13–15], and comparison of edge weights for matched orthologs [16]. Common similarity measures include calculating differences between degrees, clustering coefficients and eccentricities [17, 18], spectral signatures [19–21], and graphlet-degree signatures [22–24]. However, many of these methods provide a more generalized measure of the similarity, which can make it challenging to distinguish how specific genes actually relate to each other in a direct manner; and some of these measures can also get more and more difficult to interpret the more evolutionarily distant the species. Many of these measures may also rely on measuring the similarity between groups of genes, or modules.

One of the methods most commonly used to study the relationships between co-expression modules and to test whether a module is preserved between two different phenotypes is weighted gene co-expression network analysis (WGCNA) [8]. Although WGCNA provides insight into the conserved modules between the pairwise comparison of phenotypes, it does not provide a systematic means for comparing more than two phenotypes or networks. OrthoCluster [25] is another method that can be used to align modules in a pairwise comparison of phenotypes. However, it relies on external biological information such as one-to-one orthologs that is not always readily available specifically for non-model organisms [26]. Furthermore, different genes throughout evolution can take on similar roles and processes [27–29], and matching orthologs is not always appropriate when comparing GCNs. In contrast, it is possible to align networks by strictly using the topology of the networks. However, comparing co-expression networks topologically with alignment methods can be challenging due to their large size and the computational complexity of this type of network comparison [30]; therefore, the application of network alignment—more commonly applied to protein-protein interaction networks—to larger GCNs might be difficult.

Embedding techniques, a powerful tool in natural language processing, have also been utilized to analyse biological networks. These include matrix factorization-based methods as well as more recent neural network-based methods [31, 32]. Embedding methods provide a vectorized representation for each gene/protein and are often faster than other options, which can be critical when dealing with analysing networks [33]. Additionally, the learned embeddings are often applicable for downstream analysis as the method provides a numeric representation of the genes that can be fed into a machine learning algorithm, for example, while capturing information about how it is positioned in a network.

In this paper, we present Juxtapose, a systematic methodology for comparing multiple co-expression networks using an embedding-based approach. The proposed method does not require external biological information such as knowledge of orthologs. Juxtapose establishes both a local and global measure of similarity between networks based on their topology. Using both synthesized and real networks, we show the utility of the proposed method for comparing GCNs. Due to the lack of network alignment methods specialized for GCN alignment, we compare to PPI alignment methods that have been used or can be used to compare GCNs [7, 34]. We also compare Juxtapose to MUNK [31], which has many similarities with our proposed embedding method. However, it has been designed for PPI alignment, so it is unknown how well it performs when aligning GCNs. Furthermore, the biological relevance of the gene set enrichment analysis results after aligning real GCNs from multiple species using Juxtapose is compared to the results obtained using a common method used to compare GCNs, WGCNA.

The rest of the paper is structured as follows. Related work on GCN and PPI network analysis using embedding is described in "Related work" section. "Methods" section presents the methodology in detail and "Results" section describes the results obtained when comparing GCNs. "Discussion" section discusses the results and identifies potential caveats. Finally, "Conclusion" section ends the paper with a brief summary.

## Related work

Embedding methods stem from natural language processing (NLP), a discipline concerned with the computational methods for understanding and analysing text. An embedding for a word is a vectorized representation, i.e. a point in embedding space. Methods for learning embeddings rely on the Distributional Hypothesis, which states that words that appear in the same contexts share semantic meaning [35]. As such, semantically similar words should be mapped close to each other in the embedding space. In terms of embedding genes in the context of GCNs, co-expressed genes should be placed close together in the embedding space. When embedding GCNs, a sequence of genes can be generated by conducting a random walk on the network. These walks capture the organization of the genes in the GCN e.g., the more two genes appear in sequence, the closer their gene embedding representations will become during the model training process.

Word2vec is a neural network-based approach, which aims at learning a distributional representation of words as vectors [36]. The key components of this model are two weight matrices. The rows of the first matrix and the columns of the second matrix embed the input genes and target genes, respectively. The product of these two gene vectors is then used to get the probabilities for being a target gene, given the selected input word. A gradient descent approach can be used to learn these weight matrices by maximizing the probabilities of the true target gene(s).

Methods that extend or utilize word2vec to embed graphs such as node2vec [32] generate random walks through the networks to generate node representations. When embedding GCNs, a sequence of genes can be generated by conducting a random walk on the network. These walks capture the organization of the genes in the GCN e.g., the more two genes appear in sequence, the closer their gene embedding representations will become during the model training process. However, as node2vec was not designed to consider networks with edge weights and also does not offer strategies to create embeddings to compare across networks, we did not make use of the pipeline directly for graph embedding as it would ignore essential characteristics of GCNs.

Recent advances in machine learning have led to the development of gene representations from co-expression networks [37–39]. Gene2vec [39] and G2vec [38] are examples that utilize the word2vec [36] model originally used for natural language processing. Word2vec aims to predict the co-occurrence of a word and its surrounding words, which is called the context for that word. Analogously, in GCNs genes that are co-expressed with a given gene are considered its context. Knowing a gene and its context, these methods try to predict a gene from its context or vice versa.

Currently, these techniques have been used to predict important genes for disease within a single co-expression network. Gene2vec [39], utilizes word2vec as well as a measure of “clusteredness” of known biological pathways from MSigDB to learn gene embeddings. They used the “clusteredness” measure to encourage genes that are part of the same biological process or function to cluster together in the embedding space. They evaluated their method by its capability to cluster genes in the same biological categories, as defined by MSigDB. G2vec [38] also used word2vec to compute gene representations for identifying potential biomarkers important for cancer prognosis. Using gene expression data from cancer patients, the authors divided samples into two groups of poor and good prognosis as defined by survival outcome. For each group, they built a GCN. Then for each GCN, they generated random walks (10 walks originating from each gene). Next, these random walks were used for learning gene representations that distinguish good and poor prognosis groups. Using gene expression data acquired from TCGA transcriptomic dataset, Choy et al. implemented a two-layer neural network architecture to learn gene representations from cancer biomarker discovery [37]. To learn an association between the category of each sample and its gene expression, they trained the model to minimize the error between the predicted and actual gene expression values. They evaluated their model by its capability in clustering similar samples in the embedding space. G2vec [38] is the only method of those described above that directly compared two networks in a pairwise manner. However, combining walks from different GCNs to train a single model will convolute the gene representations as they will be a mixture of both networks. Furthermore, all of these methods utilize random walks as is traditionally done when embedding networks, which does not incorporate the weights of the edges in GCNs.

Finally, traditional techniques such as matrix factorization have shown promising results, as well as more recent manifold learning techniques to compare biological networks [40]. Fan et al. used a matrix factorization method as well as one-to-one orthologs to compare PPI networks of well-studied species, namely human, mouse, and two types of yeast [31]. Given a source PPI network, a target PPI network, and a set of homologous proteins across species, they computed diffusion kernels for each PPI network. Next, the diffusion kernel for the source species is factorized. To create protein representations that embed proteins from different species to the same embedding space, they solved a linear system of the source and target species’ diffusion kernels. The choice of the homologous proteins is essential for this approach as it can substantially affect the results of the linear system used for enforcing the embedding of multiple species to the same embedding space as it is a hard condition when solving the linear system [41].

## Methods

**Fig. 1:**
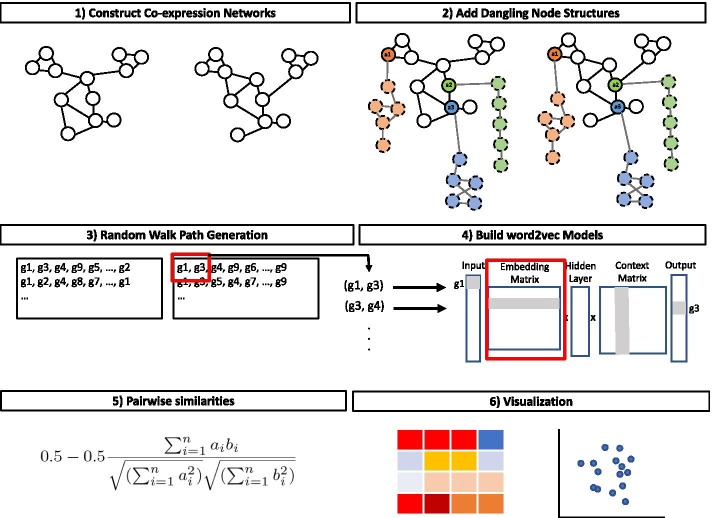
Methodology for generating joint gene embeddings from co-expression networks. **1** The co-expression networks are constructed from gene expression data. **2** Anchor genes (${a_1, ..., a_n}$) are selected as anchor nodes, which have relatively stable behaviour in the co-expression networks being compared. Dangling structures of $\gamma$ artificial nodes are added to the graphs (shown with dashed borders and edges shown in grey) with equal edge weights across the networks being compared. In this illustration $\gamma =4$. These dangling structures are connected to one of the selected anchor nodes in the original networks. **3** These networks are used to generate a set of random walks from each gene in each network. **4** The paths through the nodes are used as sentences to feed to a word2vec model, which learns informative embeddings for each gene in the networks. The model takes a gene in a network and the genes surrounding it in a path within a defined window and feeds them to a neural network that, after training, predicts the probability that each gene appears in the window around the focus gene. The process begins with a vector that contains all zeros and a 1 which represents the corresponding gene in the network. An $N \times \Vert G \Vert$ embedding matrix contains one row for every gene in the vocabulary and the number of columns equal to the embedding size *N*. Pairs of genes are used to train the model and generate a representative embedding for each gene. This newly discovered embedding vector of dimension *N* forms the hidden layer. The input gene, selected using multiplication of the embedding matrix and Input vector, is fed to the model. The multiplication of the hidden layer and the word context matrix produces the output, which will be a prediction of the most probable output gene. Then, the loss is calculated between what was expected and the gene predicted. During backpropogation, when computing the gradient of the loss function, network weights including the embeddings for all genes in the vocabulary get updated. Given a hypothetical path from a random walk ${g1, g3, g4, g9, \ldots , g2}$ and a window size of 2, *g*3 has the following input gene pairs (*g*1, *g*3) and (*g*3, *g*4) under the Skip-gram architecture of word2vec. **5** The pairwise similarity scores between genes in the embedding matrix are calculated resulting from the word2vec model. **6** The embeddings and the distances between genes in the embedding are are analysed and visualized

In this section, the following will be described: the synthetic and real datasets used, the GCN construction and methodology of Juxtapose, the evaluation, and a comparison to other approaches in the literature. An overview of the Juxtapose methodology is shown in Fig. 1.

### Data

**Fig. 2:**
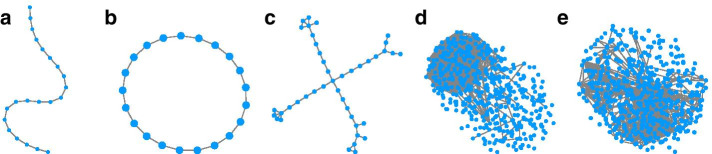
Networks used for evaluating Juxtapose. The line, circle, and cross were synthetic networks and the last two networks are a heart and brain GCN, respectively

To analyse the accuracy of the results of the proposed method, Juxtapose, we use synthetic and real GCNs. The 3 synthetic networks are shown in Fig. 2, which are only evaluated to test each method’s ability to align identical networks. The number of nodes and edges in each of these networks is presented in Table 4 of the Appendix. For the real datasets, we utilized RNA-seq data available from the GTEx project, which has expression data across many different tissues. To construct GCNs for brain and heart tissues, we used subsets of the expression data from heart ($n=200$ samples) and brain tissue ($n=200$ samples). Gene expression and sample description data were downloaded on January 18th, 2020 from the GTEx website. We used a common pipeline for preprocessing RNA-seq data [42]. The preprocessing was conducted by using Trimmed Mean of M-values (TMM) normalization, and filtering lowly expressed genes was done using the *edgeR* [43] and *limma* [44] packages in R. Several KEGG pathways in humans—see Table 1 for the list of pathways—were selected in order to construct the networks from brain and heart tissues using a method discussed in Sect. 3.2. It is hypothesized that the GCNs constructed from heart tissue samples would have more conserved networks when these GCNs are compared to each other than when compared to GCNs constructed using brain tissue samples. Similarly, brain GCNs should show more similarities to each other. Two of the networks constructed are shown in Fig. 2. Lastly, we also utilized an RNA-seq dataset originating from the prefrontal cortex of human, chimpanzee, macaque, and mouse [45] to evaluate Juxtapose using a multi-species dataset. This dataset contained 12 samples for each species. The reads of this dataset were mapped to Ensembl genome builds GRCh38, Pan_tro_3.0, Mmul_10, and GRCm38 using STAR 3.5.2 [46]. The raw counts were normalized for each species individually using TMM normalization. Any gene that did not meet thresholds was removed from downstream analyses.

**Table 1:** Gene sets used for constructing co-expression networks

ID	Description	# of Genes
hsa04260	Cardiac muscle contraction	87
hsa05410	Hypertrophic cardiomyopathy	90
hsa05010	Alzheimer disease	369
hsa05012	Parkinson disease	249
**Potential Anchor Genes**		
GO:0019725	cellular homeostasis$^*$	970

$^*$GO gene set was used to select candidate anchor genes

To construct the real GCNs, Pearson Correlation Coefficient (PCC) was calculated for each pair of genes and transformed to a value between 0 and 1 using $0.5 + 0.5PCC(g_i,g_j)$ where $g_i$ and $g_j$ are a pair of genes in a network *G*. Although PCC ranges from $-1$ (negative correlation) to 0 (no correlation) to $+1$ (positive correlation), the affine transformation above was applied to map negative correlation to 0, no correlation to 0.5, and positive correlation to $+1$. This ensures that negative correlations are separate and preserved, while allowing the values to be between 0 and 1. In order to construct the co-expression networks, a threshold of $+/-0.8$ for the original PCC values was selected before being transformed to determine whether an edge/relationship should connect a pair of genes.

### Projecting genes from different networks into the same embedding space

When networks are embedded separately, they are not necessarily going to be directly comparable. Therefore, it is useful to have pieces of the networks with a known and conserved structure. In this way, these pieces can be matched up with high confidence and can be used to align the parts of the network with unknown topology.

One strategy that has been used to jointly embed multiple PPI networks is to use a group of landmark or anchor genes [31]. However, there is an important difference when doing this procedure with co-expression networks. The selection of anchor genes when comparing co-expression networks is critical since expression relationships between some orthologous genes can vary widely depending upon the phenotypes or organisms being compared. To avoid this problem, anchor genes were selected from highly-conserved cellular processes, such as transcription and translation, which more likely contain orthologous gene positions within the co-expression network [47]. Therefore, anchor genes were selected from those annotated with homeostatic processes involved in the maintenance of an internal steady state at the level of the cell, including control of cellular proliferation and death and control of metabolic function. These genes were selected from gene sets shown in Table 1. These genes are likely to have similar connections to the rest of a co-expression network. In order to compare co-expression networks using these anchor genes, we propose a method to embed genes in the same embedding space.

Figure 1 illustrates the steps for preparing two co-expression networks $G_1$ and $G_2$ for embedding and these steps are described as follows. First, the anchor genes need to be selected, ${a_1, ..., a_n}$, that are present in the networks that will be embedded. We use anchor genes to provide a base for model evaluation. Since anchor genes are expected to be aligned across species, if we have the same graph structure attached to these genes, the structures also are expected to be aligned across GCNs. Thus, different synthetic structures are created and the same structure is added to matching anchor genes across networks. Hereafter, we refer to such a synthetic structure as a dangling structure. For a selected anchor gene, the dangling structure created is a random sparsely connected graph. The number of nodes for a dangling structure $\gamma$ ($\gamma \in {\mathbb {N}}$) is a hyperparameter for the model representing the number of nodes in a dangling structure. The nodes in the dangling structure are connected using $15\%$ of all its potential edges. If the resulting dangling structure is not a connected graph, a minimal number of edges required for making the dangling structure a single component is randomly added to the dangling structure. All edges in the dangling structure are assigned a weight equal to 1.

**Fig. 3:**
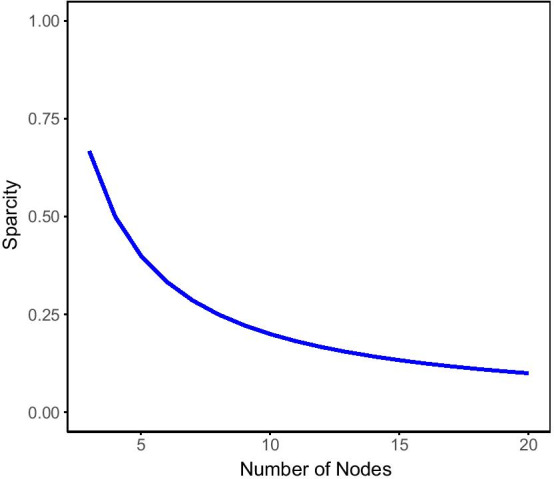
Line graph illustrating the required minimum sparcity levels in order to create a connected sythetic structure to attach to the real portions of the gene co-expression networks. The x-axis shown the number of nodes used to construct the synthetic structure and the y-axis shows the minimum level of sparcity (numbers of edges connecting nodes of the synthetic structure) required to make a connected graph, i.e., there is only one component that includes all of the nodes in the synthetic structure

As each synthetic structure should be a connected component of the graph, the minimum sparsity requirements for *n* nodes is equal to $\frac{2}{n}$. Also, to avoid disconnected components, even if the choice of sparsity is under the minimum value required to make a connected structure, then the proposed approach will add connections until a connected component is achieved. However, it should be insured that all components are unique structures after construction. Figure 3 shows the minimum levels of sparsity required in order to make synthetic structure that could potentially be connected. The rationale behind using a sparse artificial network is that nodes in dense networks are topologically similar. Therefore there is not much topological variation that can be used for model evaluation, and any naive embedding that maps all nodes to almost the same value could be considered a reasonable solution. Therefore, we use sparse graphs to provide a better estimate of model capability in encoding topological variation among nodes.

### Generating walks and model training in Juxtapose

Walk generation was performed in Juxtapose by converting the weights of the GCNs to a probability of travelling through the edges connecting genes. The higher a correlation value, the more likely a walk would travel through the edge. In order to handle large real networks in Juxtapose, translation from gene names or Ensembl IDs to integer values was performed in order to give the method the power to generate a large number of walks quickly. This translation generates a JSON file to make it convenient to convert integer values back to gene name or IDs for visualization and for interpreting results.

In order to generate gene embeddings, *gensim* version 3.8.3 was utilized. A word embedding was trained by maximizing the probability of gene co-occurrences in context, i.e., only a few genes apart in a single walk. Analogously, we defined the context of a gene by the other genes that are co-expressed with it. An $N \times \Vert G \Vert$ embedding matrix is randomly initialized and contains one row for every gene in the vocabulary and the number of columns equal to the embedding size *N*. This newly discovered embedding vector of dimension *N* forms the hidden layer. An input gene is fed to the model in order to generate predicted output genes, meaning the genes that are most likely to follow the input gene in the generated walks within a set window. The multiplication of the hidden layer and the word context matrix produces the output, which will be a prediction of the most probable output gene. Then, the loss is calculated between what was expected and the gene predicted. This process continues with all of the generated walks.

The parameters used to generate the embeddings for genes in each dataset are provided in Table 5 of the Appendix. We rely on our ability to generate training data from the GCNs by using more walks per gene rather than increasing the number of training epochs or iterations, which can cause overfitting [48]. This is often not possible for many applications since the amount of training data can be limited. However, in the context of GCN, one can extract a large dataset of random walks. This has the benefit of (1) providing a better representation of a co-expression network by having a large number of random paths and (2) not needing to repeat the training for a large number of epochs. Indeed, our model used only 1 epoch, and it generated a large number of random paths from the entire network. Instead of, for example, using 10 walks per gene and iterating over this 100 epochs we use 1000 walks per node with 1 epoch.

### Measuring similarity of embedded genes, aligning networks, and measuring network similarity with Juxtapose

One local and one global measure of similarity between genes was used in order to compare the genes of two co-expression networks. The local similarity measure utilized between all pairs of gene vectors between the two networks was cosine distance, which measures the cosine of the angle between them. Cosine distance between pairs of genes was calculated as shown in Equation 1, where $a = (a_1, \ldots , a_n)$ and $b= (b_1, \ldots , b_n)$ are the gene vectors/embeddings.

1 $$\begin{aligned} \text {cos distance}(a, b) = 0.5 - 0.5 \frac{\sum _{i=1}^n a_i b_i}{\sqrt{\left( \sum _{i=1}^n a_i^2\right) } \sqrt{\left( \sum _{i=1}^n b_i^2\right) }} \end{aligned}$$

One advantage of cosine distance is that it has low computational complexity, where only the non-zero dimensions of the gene vectors need to be considered. Furthermore, cosine distance tends to be effective at estimating the distance between vectors when they have a high dimension [49]. Indeed, as the structure of GCNs can be quite complex, and the number of genes in these networks is often in the thousands, the gene embeddings may require a high dimension in order to represent their position in the GCNs accurately.

With this local distance measure between genes of different networks, it is then possible to match genes from one network to the other. A matching algorithm (formally, on bipartite graphs) is an algorithm that takes two lists of elements where there is a distance between every element of one list to every element of the other, and constructs a “matching” between the two lists—a matching associates every element of one list with exactly one element of the other list in such a way that each element only gets associated once—and it does so in such a way that the sum of the distances matched is minimal over all possible associations. The Hungarian algorithm is a well-known matching algorithm that runs in polynomial time complexity. The matching constructed by the algorithm is mathematically guaranteed to be optimal, and have the smallest sum of matched distances [50]. In our case, the two lists are the genes in the two GCNs being compared, and the distance between pairs of genes of the two networks being compared is the cosine distance. Thus, the Hungarian algorithm in the scikit-learn Python library [51] is used to create a type of global similarity by producing the best global alignment (matching) of genes in two networks based on their pairwise angular distance. This matching not only provides an optimal association (or alignment) between genes of the two networks, but the sum (or equivalently, average) of the matched distances provides a global similarity score between the networks being compared. As there was a distance calculated between each pair of genes, groups of genes that have similar patterns of distances can also be grouped using a biclustering method. This can also be overlaid with other biological information for other downstream analyses.

Biclustering was utilized in order to discover groups of genes that have similar distances to each other as well as similar differences to other genes. Spectral Biclustering assumes a checkerboard structure where the same gene can belong to multiple biclusters [52]. The rows and columns of a matrix with this structure may be partitioned so that the entries of any bicluster in the Cartesian product of row clusters and column clusters are approximately constant. For instance, if there are two row partitions and three column partitions, each row will belong to three biclusters, and each column will belong to two biclusters. Biologically, genes may be involved in different biological processes and have different patterns of distance between genes. This method of biclustering was used since the biclusters generated provide clusters of genes that have similar distances from a gene of interest to different degrees. Gene set analysis was performed on the resulting biclusters on the non-simulated networks using WebGestalt [53].

### Evaluation of Juxtapose

Results from two common methods of graph alignment, IsoRankN [20] and MAGNA++ [54], as well as MUNK [31] and WGCNA [8] were compared to the results of the gene embedding method Juxtapose, where appropriate. IsoRankN and MAGNA++ were evaluated based on their ability to align the nodes of equal or similar networks and the information captured by their similarity scores. Real networks for brain and heart were also compared to each other in order to compare similarity results from Juxtapose to the results from IsoRankN, MAGNA++, and MUNK. The percentage of correctly aligned genes was determined by measuring the proportion of genes with corresponding gene names in each aligned GCN that were matched together in an alignment. Juxtapose was further evaluated with large, real GCNs from multiple organisms to demonstrate its ability to handle various GCNs with different genes as well as to assess the method from a biological perspective. WGCNA was compared based on the conserved modules identified in pairwise comparisons between GCNs of real large networks from the prefrontal cortex of multiple species.

## Results

The following sections present the results of network comparison using Juxtapose. Section 4.1 reports the results of comparing identical synthetic and real GCNs using Juxtapose and comparing these results to PPI network alignment methods IsoRankN and MAGNA++. Section 4.2 includes the comparison of GCNs constructed using different subsets of samples from brain and heart tissue samples and compares the results of Juxtapose to IsoRankN, MAGNA++, and MUNK. Finally, Sect. 4.3 applies Juxtapose to large GCNs constructed from multiple species and compares to WGCNA.

**Table 2:** Percentage of matched genes in self-aligned networks reported for MAGNA++, IsoRankN, and Juxtapose

	MAGNA++				IsoRankN				Juxtapose
	alpha 0.50	alpha 0.95	alpha 0.50 with 50% bitscores	alpha 0.50 with 100% bitscores	alpha 0.50	alpha 0.95	alpha 0.50 with 50% bitscores	alpha 0.50 with 100% bitscores	N/A
Line	0	0.10	0.52	1.0	0.24	0.19	0.0	1.0	1.0
Circle	0	0.14	0.57	1.0	0.33	0.29	0.0	1.0	1.0
Cross	0.24	0.02	0.52	1.0	0.29	0.19	0.83	1.0	1.0
Heart	0.93	0.93	0.99	1.0	0.16	0.04	0.71	1.0	1.0
Brain	0.33	0.55	0.99	1.0	0.18	0.07	0.82	1.0	1.0

The alpha values indicate the balance between node similarity and edge similarity (MAGNA++) or the balance between topological similarity and sequence similarity (IsoRankN). When no percentage of bitscores is provided, the algorithm was not provided with informative bitscores i.e. the match between any genes was equally likely. When 50% of bitscores were provided, 50% of the genes had the highest bitscore provided for the real match between the genes of both networks. When 100% of bitscores were provided, 100% of the genes had the highest bitscore provided for the real match between the genes of both networks. N/A is given for the settings in Juxtapose as no bitscore file is provided and no alpha value is provided to the tool

### Alignments of identical networks

Table 2 indicates the percentage of correctly matched genes for IsoRankN, MAGNA++, and Juxtapose. Both IsoRankN and MAGNA++ have a parameter (alpha) that for IsoRankN indicates the extent to which network topology is used to make the network alignment—where 1 is completely topology based—and MAGNA++ has a alpha value that balances between node and edge conservation. Furthermore, we provide these methods with different degrees of knowledge about known node matches between the networks in the form of (sequence similarity) bitscores. If 100% of the bitscores are provided, this means that the bitscores clearly indicate which matches are the most appropriate matches between nodes e.g. the corresponding genes between networks have a value set to 1 and the remaining node matches are set to zero. Juxtapose does not use any sequences and therefore matching does not take sequence similarity into account and is purely topologically based. The performance of the alignment methods was measured based on their ability to align the corresponding genes in the networks compared e.g., $gene_1$ in a GCN correctly aligned to $gene_1$ in a duplicate version of the GCN would be counted as a match.

IsoRankN and MAGNA++ were able to match all of the corresponding nodes of the two networks only when provided with the known matches in the form of high sequence similarity i.e., high bitscore values. This is in agreement with an observation also reported by [19] that including sequence information improves the performance significantly. These methods sometimes struggled with aligning the structures that had symmetry such as the line and circle synthetic networks if artificial bitscore matches were not provided to the algorithms. IsoRankN had relatively higher scores than MAGNA++ for the synthetic networks when no biological similarity was used during the alignment process. The exception of low performance without bitscores was that MAGNA++ was able to align the heart GCN with 93% of the nodes matched correctly. However, Juxtapose reported the most appropriate matches compared to the results of these two alignment methods, perfectly aligning the networks in every case. This is especially noteworthy given that Juxtapose also did not require any known matches between genes to be provided in terms of external biological information such as sequence similarity and was mainly using network topology to align the networks. Juxtapose was able to align the true matches only using the cosine distance between gene embeddings followed by the Hungarian algorithm to determine the match with the lowest cost.

### Alignment of different networks

First, to assess the choice of hyperparameters, we compared the average distance between anchor nodes and random genes to ensure that the selected anchors used to build synthetic structures into the real networks were appropriate for the analyses. We generated a selection of 1000 sets of random genes of equal size to each synthetic structure and compared the sum of the similarities between matched genes in both groups. The distances between the anchor nodes was significantly less than the distances between nodes in the random groups of genes (p-value < 0.001). Therefore, the hyperparameters selected as well as the anchor genes were determined to be appropriate for the following comparisons.

**Fig. 4:**
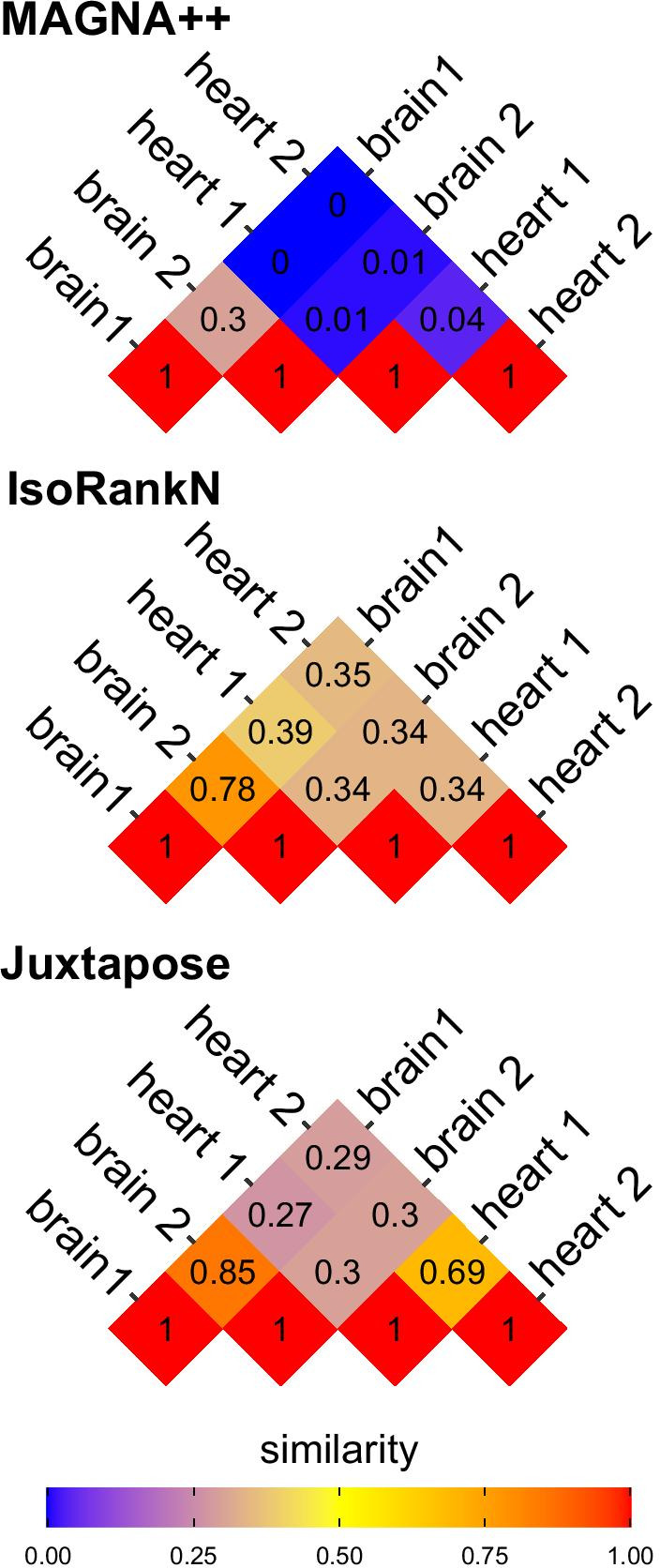
Pineplot illustrating the proportion of genes matched between heart and brain networks compared using (from top to bottom) MAGNA++, IsoRank, and Juxtapose. The pineplot was constructed using the *pineplot* R package v 0.0.9 [60]

**Fig. 5:**
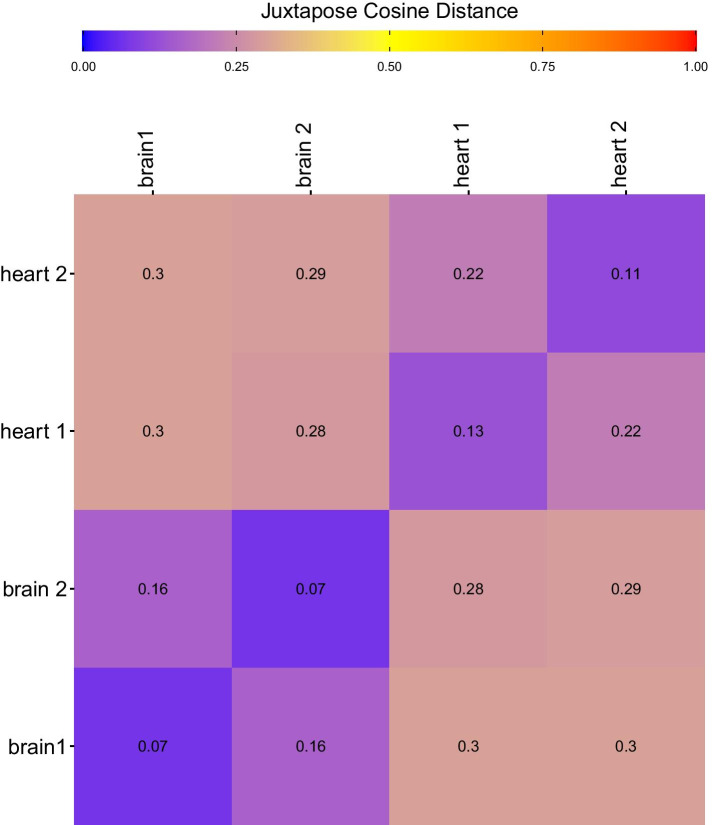
The global cosine distances between heart and brain networks compared using Juxtapose. A distance closer to 1 indicates the networks are less similar and a distance closer to 0 means the networks have more similarity

Next, to assess the alignment of different networks, we generated 2 replicate GCNs from subsets of non-overlapping brain and heart samples. As such, these replicates generated similar, but not equivalent, network structures. Each replicate was compared in a pairwise fashion using Juxtapose, and the proportion of correctly matched genes between different networks constructed from the same tissues as well as between altogether different tissues was recorded and visualized in Fig. 4. The proportion of matches was significantly higher (0.69 and 0.85) when comparing the same tissues vs. when comparing between tissues where the proportion of matches was never more than 0.3. Further, the global similarity values for Juxtapose are shown in Fig. 5. Juxtapose reported global distance scores around 0.3 between tissues; i.e., GCNs that are less similar to each other, for the GCN comparisons made between brain and heart compared to the distances reported for comparisons between GCNs from the same tissue type. Also, the heart GCNs result in global distances that were higher than the ones reported for brain networks. This likely has to do with the number of edges in the heart networks that form a “hairball” topology. Although the most similar genes tend to be the corresponding genes in the other network, the distance between the genes is much higher.

**Fig. 6:**
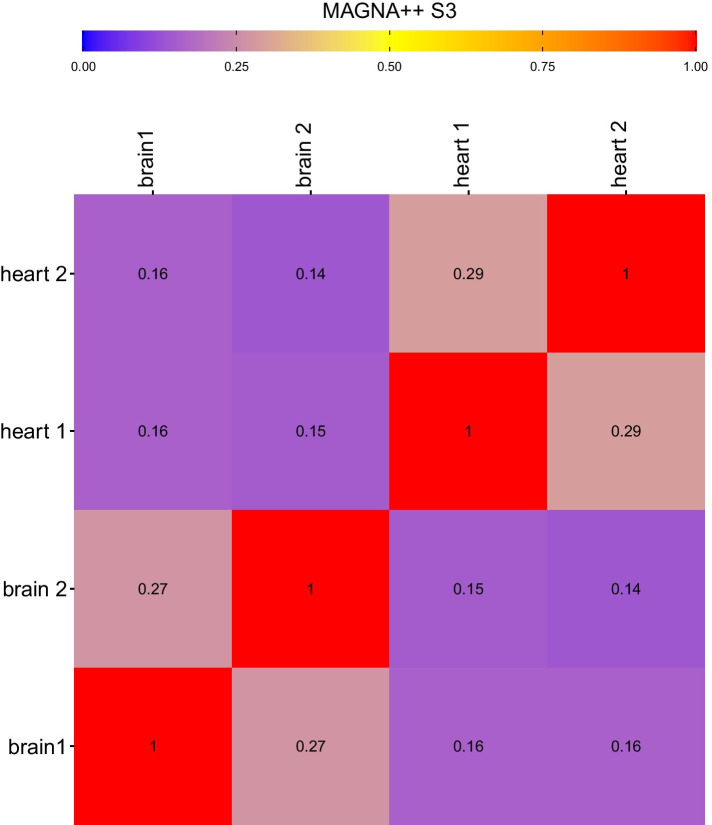
The S3 similarity score between heart and brain networks compared using MAGNA++. Using MAGNA++, a value closer to 1 indicates the networks are more similar and a distance closer to 0 means the networks are more distant. Bitscores were provided to each method and the alpha value was set at 0.5

**Fig. 7:**
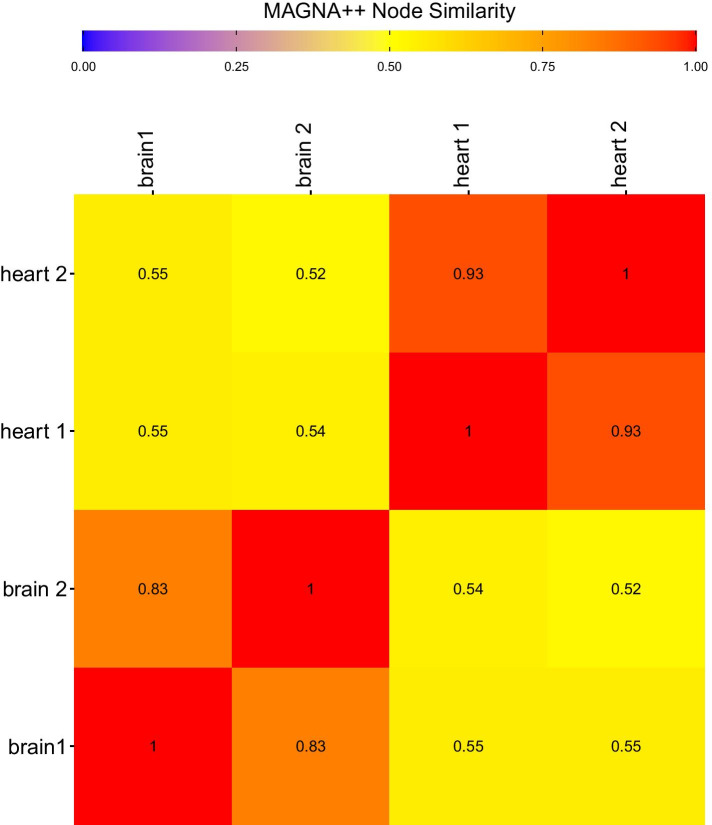
The node scores (NS) between heart and brain networks compared using MAGNA++. Using MAGNA++, a value closer to 1 indicates the networks are more similar and a distance closer to 0 means the networks are more distant. Bitscores were provided to each method and the alpha value was set at 0.5

Similarity measures for IsoRankN and MAGNA++ when all bitscores for matched genes are provided are shown in Figs. 4, 6, and 7. The proportion of matched nodes did not agree with the similarity between brain and heart networks when using IsoRankN or MAGNA++. The two brain networks were reported as most similar by IsoRankN after the self comparisons, which is reasonable. The next most similar alignment occurred when comparing a brain network to a heart network (0.39). The comparison between the two replicate heart networks is one of the lowest scores (0.34). MAGNA++ had a relatively low percentage of matched nodes between networks. However, MAGNA++ has an S3 score and node score shown in Figs. 6 and 7 that reflect the similarity of the networks and it is usually comparable to Juxtapose (but again, MAGNA++ is using bitscores while Juxtapose is not). This score penalizes GCN alignments that map denser network regions to sparser ones or alignments that map sparser network regions to denser areas. However, the proportion of matched nodes remains relatively low and the similarity when comparing heart networks to brain networks is much lower than the scores reported by Juxtapose even though the genes present in these networks and their structures overlap significantly.

We also compare MUNK to Juxtapose using the heart GCN replicates. The synthetic networks constructed in Sect. 4.1 were not used to compare to MUNK as there are some characteristics that make them unsuitable input to MUNK. The method has been designed for PPI networks that are sparse, unweighted, and directed. Some of the limitations of MUNK for co-expression networks include the following items.

- Requires networks to be directed
- Does not utilize edge weights
- Removes nodes with a degree less than 2 so their representation will never be learned
- Requires one-to-one orthologs mapping to perform alignment of the networks
- Only analyzes the largest connected component, so if there are two connected components, it will only take into account the largest one and the rest of the genes are lost before the comparison is made

We take the upper triangular correlation matrix of the heart and brain replicate GCNs to form a directed version of the network and remove the weights from the edges. The largest connected component of the networks were 154 and 153 nodes, respectively so these were the components used to make the alignment. Since MUNK uses a linear mapping, it was capable of producing an almost exact match between duplicate heart networks (98.7%). However, MUNK was only able to align 1 (<1%) of the genes successfully between the heart and brain networks where Juxtapose was able to align roughly 30% of the genes between these networks. This may be due to the ability of Juxtapose to consider the edge weights as well as the undirected nature of the networks, allowing the random walks to pass through an edge in either direction and learning more informative embedding in the context of GCN comparison compared to PPI comparison. In this way, Juxtapose can identify genes with similar connectivity in different networks more successfully in the context of GCN comparison.

**Fig. 8:**
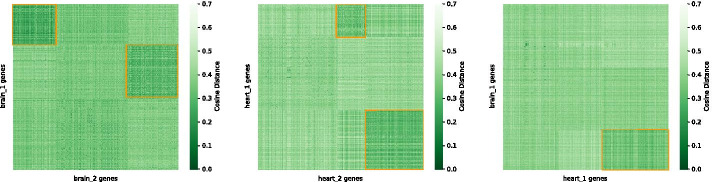
Biclustering results for the cosine distance matrix for one replicate of the heart GCNs and one replicate of the brain GCNs. Dark green indicates less distance between nodes while light green or white indicates nodes are more distant from each other. The orange boxes indicate groups of genes that have lower local cosine distances, indicating more conservation between these genes in the GCNs being compared

Figure 8 shows the result of biclustering the cosine distance matrix comparing the heart and brain networks with spectral biclustering. The brain GCNs had the most similarity overall, with the most conserved bicluster containing the lowest cosine distances containing genes that were mostly from the Alzheimer disease and Parkinson’s disease KEGG pathways (96% of the genes in the top left bicluster were part of the brain disease pathways, and the next most conserved module contained 25% of the heart-related genes). The heart GCNs, on the other hand, resulted in the most conserved bicluster containing a large portion of genes from the KEGG heart-related pathways (30% of the genes were from the heart KEGG pathways in the most conserved bicluster in the bottom right corner, and 26% of these genes were present in the next most conserved bicluster). Furthermore, when comparing one of the heart GCNs and a brain GCN, the genes that were closest to each other based on cosine similarity were mostly from the Alzheimer disease and Parkinson’s disease KEGG pathways in both the brain and heart network as opposed to genes specific to the heart KEGG pathways. In the bicluster with the smallest distances between genes, the heart GCN had 78% of the genes from the brain KEGG pathways and 19% from genes shared in both the heart and brain KEGG pathways. Only 3% were strictly from the heart KEGG pathways.

### Prefrontal cortex multi-species

Finally, we utilized Juxtapose to compare large, real GCNs from different species. Bozek et al. observed an acceleration of metabolite concentration differences among tissues that were confirmed by expression-level differences in corresponding genes in the prefrontal cortex of the brain and in skeletal muscle [45]. They also predicted that these rapid changes might reflect parallel mechanisms in human evolution. We attempt to find evidence of differences in the gene expression regulating the metabolome by constructing GCNs from the data generated by Bozek et al. and comparing the networks using Juxtapose. In the biclusters, we identified groups of genes that were far apart—and thus possible candidates for adaptation with respect to mammalian brain metabolomics—between the species which we selected and performed over-representation analysis to identify modules with enriched KEGG pathways.

Juxtapose was able to identify multiple biclusters with enrichment for KEGG pathways associated with metabolism. Many of these biclusters also had relatively low cosine distances between human and the other three species, suggesting differences in topology in portions of the networks. For example, when comparing human and chimpanzee GCNs biclusters with cosine distances over 0.5, i.e. relatively distant, had enriched terms including Amphetamine addiction, Dopaminergic synapse, and Thyroid hormone signaling pathway, which were reported in the paper by Bozek et al. and include genes that are important regulators of growth, development and metabolism. The biclusters that showed the most difference between these species also included enrichment for choline metabolism. All the compared species had biclusters with high cosine distances (indicating less similarities in these parts of the networks from a topological perspective) in biclusters containing KEGG pathways including Valine, leucine and isoleucine degradation, Inositol phosphate metabolism, Tryptophan metabolism, Pyruvate metabolism, beta-Alanine metabolism, and Propanoate metabolism, beta-Alanine metabolism, some of which were also identified by Bozek et al. [45]. Glutamatergic synapse and Aminoacyl-tRNA biosynthesis were terms enriched in a bicluster that was slightly more similar i.e., had lower cosine distances between these two species. These results are in support of human-specific metabolic divergence as found by Bozek et al. [45]. From the global cosine distance score, macaque and mouse were the most distant from human with global cosine distances of 0.41 and 0.40, respectively and human was the most similar to chimpanzee with a global cosine distance of 0.34. These results are presented in Table 3. As the global cosine distances are relatively low for all of the species, these results suggest that there is a lot of conserved portions of the networks as well. These global cosine distance results are also supported by the WGCNA results described below. This observation supports that the global cosine distance scores reported by Juxtapose can also be informative when analysing large GCNs. The biclustering enrichment analysis results for each species are presented in the Supplementary Materials. Below, we describe the similarities and differences between the results discovered using Juxtapose and the well-established GCN analysis tool WGCNA.

**Table 3:** Global cosine distances reported by Juxtapose when comparing prefrontal cortex GCNs from human, chimpanzee, macaque, and mouse

	human	chimpanzee	macaque	mouse
human	0	0.34	0.41	0.40
chimpanzee		0	0.36	0.36
macaque			0	0.35
mouse				0

For the global distance measure, a distance closer to 1 indicates the networks are less similar and a distance closer to 0 means the networks have more similarity

The results of the WGCNA analyses are presented in Figure S1 and S2 of the Supplementary Materials. The hierarchical clustering results showed similar patterns for human and chimpanzee gene modules with the macaque clustering appearing the most distinct with one cluster containing a large proportion of the genes. Mouse, on the other hand, had more visual similarity with the human and chimpanzee clustering results. However, the Zsummary scores were relatively low in human versus mouse compared to human versus the other two species (chimpanzee and macaque). The mouse transcriptome being the most distinct from the other three species agrees with the original publication, which concluded that the human metabolome underwent greater change in a shorter period of time than the mouse metabolome did over the 130 million years separating mice from the common ancestor of humans, chimpanzees, and macaques [45]. Mouse is also the most phylogenetically distant from human among these species. We selected modules that showed little to no evidence of preservation (Zsummary < 2) and performed over-representation analysis to identify modules with enriched KEGG pathways. The cyan and pale turquiose modules were the only modules with low preservation that were enriched for any KEGG pathways. Pancreatic secretion, Protein export, Longevity regulating pathway, and Oocyte meiosis, were enriched in the pale turquoise module while the cyan module was enriched with Legionellosis. Of these enriched terms, Pancreatic secretion and Oocyte meiosis are the only enriched terms in the low preservation modules that overlap with the terms reported by Bozek et al. as enriched in the human–specific concentration profiles in the prefrontal cortex. In fact, most of the enriched pathways show up in the highly conserved modules such as the turquoise module, which includes enriched terms such as Amphetamine addiction, Cocaine addiction, Dopaminergic synapse, Chemokine signaling pathway, Aminoacyl-tRNA biosynthesis that were identified in the original publication. This suggests that although the expression levels of the genes in these clusters may be quite different, they have not changed as much in terms of their co-expression with other genes.

## Discussion

This paper introduced Juxtapose, a tool for comparing the topology of GCNs utilizing a gene embedding approach. One benefit of using Juxtapose as a means of comparing networks is that no knowledge is required about the genes themselves from a biological perspective in order to make a relatively good alignment compared to other alignment methods. Using this embedding method, it is easy to identify not only the best matches with a gene in a corresponding network, but also observe the similarity of a gene to all other genes in the network as well with the local cosine distances. In this way, it is possible to identify areas in the networks that are unambiguous matches (highly conserved) vs. more ambiguous matches (good matches to many genes). It also allows for orthologs that are not strictly one-to-one or functional orthologs to be analyzed to get a more complete picture of the similarities and differences between GCNs, which is a particularly attractive feature for evolutionary studies.

Juxtapose appears to outperform existing alignment-based methods for identifying similar nodes/genes. Indeed, even when aligning artificial networks with unique structures, the typical alignment-based methods performed poorly without prior knowledge of gene similarity. This makes these methods not as informative for aligning co-expression networks. Our method was able to identify the known matches between the identical networks without knowledge of gene similarity. We show that the score of the known matches is also the minimum score one can get by employing the Hungarian algorithm for making the global alignment of the nodes in each network. Therefore, Juxtapose was able to outperform these alignment methods even though its intended purpose is not necessarily to align corresponding nodes in the graphs, but to obtain a measure of similarity between all genes being compared between GCNs.

Juxtapose also outperforms MAGNA++, IsoRankN, and MUNK for aligning different GCNs to one another. MAGNA++ and IsoRankN are only able to achieve comparable results to Juxtapose when they are provided knowledge of the similarity between genes based on biological information such as bitscores. Juxtapose has no such requirement. MUNK also requires some knowledge of orthologs for landmark selection; however, the requirements that likely cause the method to perform more poorly on GCNs compared to PPI networks are that it requires a directed network as input, and it cannot utilize the edge weights to make the alignment. Furthermore, MUNK only operates on the largest connected component of the graph, which may lead to the similarities between some genes not being calculated. Juxtapose, on the other hand, is able to report both local and global distances or similarities between all genes in a GCN.

Another benefit of the proposed methodology is that since it relies on probabilistic walks through the co-expression networks, differences at the level of gene expression or correlation across species do not require normalization across the networks being compared. Normalization tends to be a significant challenge in gene expression analysis, especially when the data has been sequenced in different batches, labs, etc. However, needing to apply multiple types of normalization can actually obscure real signal in the data as none of them work perfectly [55]. Furthermore, there may be unknown factors that require normalization that are missed [56]. Methods such as IsoRank and IsoRankN have been utilized for comparing co-expression networks, but they were not originally designed for analysing these types of networks. Therefore, assumptions have to be made about the data that may limit the analyses of GCNs. MUNK also has assumptions that may limit the analysis of GCNs, so although these methods may work well for analysing PPI networks, more methods that are specifically designed for comparing GCNs are required. Juxtapose is much more adaptive for networks that require weights on the edges compared to many alignment strategies originally designed for PPI networks.

We also demonstrated that the local cosine distances comparing genes from different GCNs is biologically informative. The biclustering results of the heart and brain GCNs revealed that there was more conservation observed between the genes from the brain-related KEGG pathways. The genes of the heart pathways were more conserved in the heart GCN compared to the brain GCN. This also supports the hypothesis that more conservation would be observed in genes important for regulating processes in the brain, as is supported or suggested in the literature [57].

Ultimately, the goal is to utilize this method in order to compare networks constructed from homologous samples in different species, so we also applied this method to networks constructed from gene expression data from different species. These results could indicate genes that show more evidence of constraint or adaptation between the networks compared. The biclustering results analysing the local cosine distances between human, chimpanzee, macaque, and mouse identified modules of genes that contained enriched KEGG pathways related to metabolism. Also, these modules of genes tended to have high cosine distances, suggesting that these portions of the GCNs across species were less conserved. Juxtapose was also able to identify more terms specifically related to different metabolites compared to both WGCNA and the hierarchical clustering performed by [45] while also having results that agreed with the observations made by [45] as well. These results show that Juxtapose can produce results that complement WGCNA results while making it easier to determine the distances or similarities for all pairwise comparisons between modules of genes.

One consideration when dealing with very dense networks that have thousands of genes using Juxtapose is time and memory bottlenecks. For example, for a network as dense and large as the multi-species dataset, a large amount of memory is ultimately required given the number of walks used for training the models. The number of walks and their length ultimately informs how much memory is going to be required. Other hyperparameters of the model training do not have much influence on memory requirements. However, the sliding window parameter used to train could significantly impact biological interpretations, especially for co-expression networks. Since the networks tend to have a lot of false positives, increasing the window in effect is similar to creating more direct edges between genes that probably do not have a direct relationship. As future work, we suggest measuring how much changing this particular hyperparameter impacts the resulting biological interpretation.

**Fig. 9:**
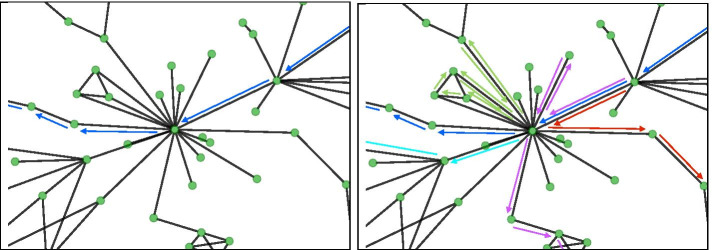
Visualization of a simple example of probabilistic random walks demonstrating the importance of walk number to avoid overfitting when training a model with gene co-expression networks. A small number of walks is represented by the image on the left, where the blue arrows indicate a walk that is going through a hub gene (center node) in the network. A larger number of walks through this same node is shown on the right. Each arrow colour indicates a separate walk. Assuming that all of these edges have comparable edge weights, if we have a small number of walks that travel through this node, which is more likely the fewer/shorter walks we make, than this gene will not be well represented in the final model, no matter how many iterations/epochs are made during the training. This challenge would become even greater when hub genes are also connected to each other which increases the possibilities for distinct walks immensely. Densely connected nodes tend to be a characteristic of gene co-expression networks, so the number of walks is an important consideration

The choice of the number of walks selected in order to train the word2vec models is also dependent on the density of the networks being analysed. Consider a network that is not sparse, which tends to be the case with gene co-expression networks. For example, generating ten walks from each node in the network is less likely to be an accurate representation of genes highly connected to many other genes. In Figure 9, we show a simple example of this scenario. The fewer/shorter walks made, the worse the representation will be for dense portions of the network in the final model, no matter how many iterations/epochs are made during the training. This challenge would become even greater when hub genes are also connected to each other, which increases the possibilities for distinct walks immensely.

One limitation is the need to confirm that the dangling pieces of the network are spread out in different areas. Future work may also include exploring different updates to the loss function. If they were incorporated directly into the gene expression data, this would not be an issue and will be a goal of future research. There are also newer state-of-the-art embedding strategies in NLP that use Transformers available that can be adapted to embed networks such as BERT [58], ELMO [59] etc. It would be interesting to apply these context dependent methods in future research, particularly with biological networks that have direction to their edges.

## Conclusion

Gene co-expression networks are not easily comparable due to their complex structure. In this paper, we proposed a python-based tool and similarity measures that can be utilized for comparative co-expression network analyses. A word embedding strategy commonly used in natural language processing was adapted and utilized in order to generate gene embeddings based on walks made throughout the gene co-expression networks.

The utility of Juxtapose was demonstrated in scenarios such as comparisons between species and tissues. Synthesized datasets, RNA-seq datasets from GTEx, and a multi-species experiment of prefrontal cortex samples from the Gene Expression Omnibus (GEO) were used to demonstrate its ability to embed the nodes of synthetic structures in the networks consistently while also generating biologically informative results in real networks. Furthermore, Juxtapose is able to successfully align GCNs without relying on known biological similarities and enables post-hoc analyses using biological parameters, such as orthology of genes, or conserved or variable pathways.

### Supplementary Information

**Additional file 1.** Supplementary Figures S1–S2 illustrating WGCNA hierarchical clustering results and module preservation statistics of gene co-expression networks constructed using prefrontal cortex samples, and Supplementary Table S1 containing lists of genes present in the highlighted biclusters of Figure 8 with the smallest cosine distances.

## Data Availability

A development version of the software used in this paper as well as datasets are available at https://github.com/klovens/juxtapose

## Appendix

See Tables 4 and 5.

**Table 4:** Node and edge information for networks used for evaluation

Network	# of Nodes	# of Edges
**A**	21	20
**B**	21	21
**C**	42	47
**D**	484	2862
**E**	484	742

**Table 5:** Parameters used for generating embedding for each GCN evaluated

	Synthetic Networks	Heart and Brain Networks	Prefrontal Cortex Networks
walk_per_node	1000	1000	1000
walk_length	20	50	30
n_iter	1	1	1
n_workers	20	20	10
embd_dim	30	150	150
window	2	2	2
min_count	2	2	2
negatives	5	15	50
alpha	0.0025	0.0025	0.0025
min_alpha	0.001	0.001	0.001

## Abbreviations

GCN: Gene co-expression network
GEO: Gene Expression Omnibus
GTEx: Genotype-Tissue Expression
KEGG: Kyoto Encyclopedia of Genes and Genomes
PPI: Protein-protein interaction
MSigDB: Molecular Signatures Database
MUNK: Multi-species network kernel
NLP: Natural language processing
PCC: Pearson correlation coefficient
WGCNA: Weighted correlation network analysis
TCGA: The Cancer Genome Atlas
