# Supplementary material for: Juxtapose: a gene-embedding approach for comparing co-expression networks
Source: BMC Bioinformatics. 2021 Mar 16;22:125. doi: 10.1186/s12859-021-04055-1 (PMC7968242; doi:10.1186/s12859-021-04055-1)
Supplement: Supplementary file 1 — Additional file 1. Supplementary Figures S1–S2 illustrating WGCNA hierarchical clustering results and module preservation statistics of gene co-expression networks constructed using prefrontal cortex samples, and Supplementary Table S1 containing lists of genes present in the highlighted biclusters of Figure 8 with the smallest cosine distances. [file 12859_2021_4055_MOESM1_ESM.pdf]

Supplementary Materials

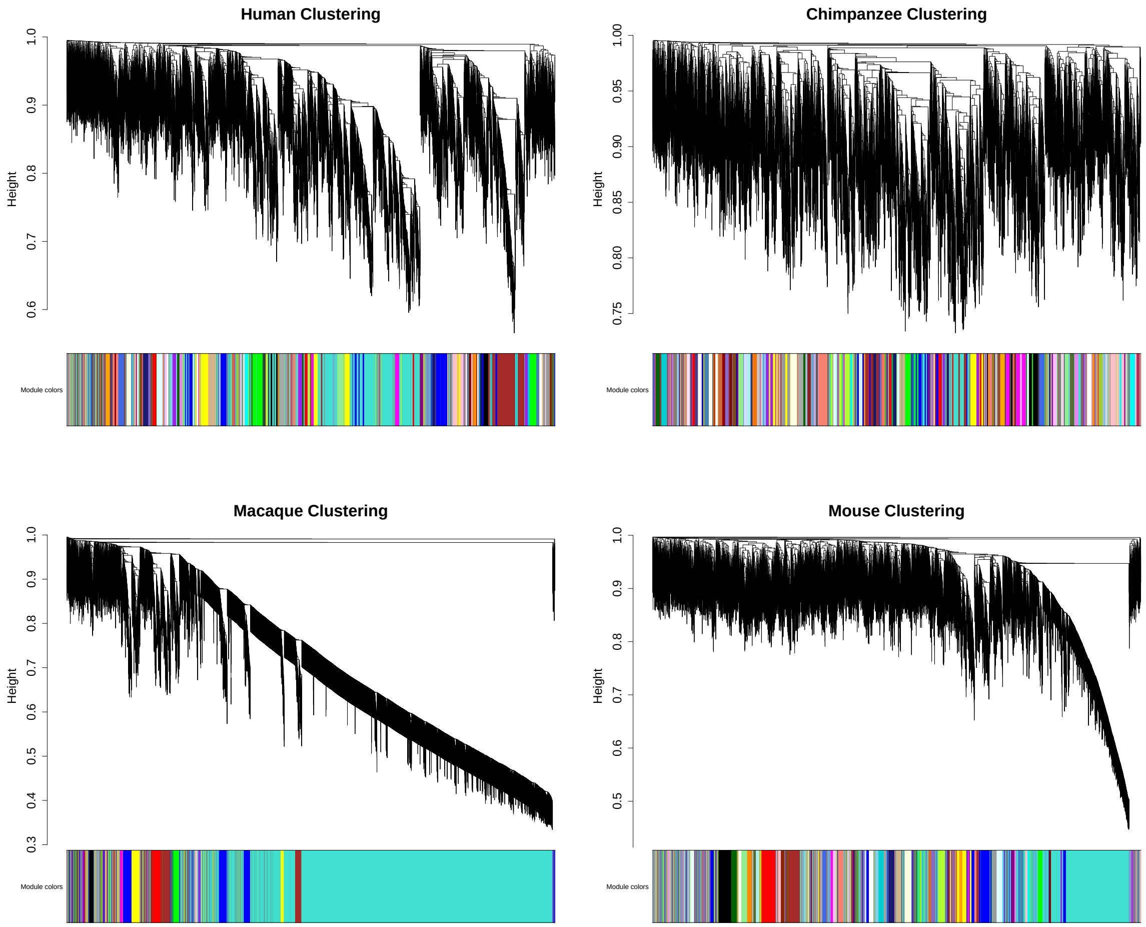

Figure S1: Hierarchical clustering results of gene co-expression networks constructed using prefrontal cortex samples from human, chimpanzee, macaque, and mouse. To generate the networks, a power of $\beta = 8$ for soft thresholding that resulted in a scale-free network topology. The same genes used in the analysis using Juxtapose were considered for this run of WGCNA. The clustering merge height was set to 0.50 to merge to generate the clusters shown in the dendrogram and module colour images (bottom). The minimum module size allowed was 30 genes.

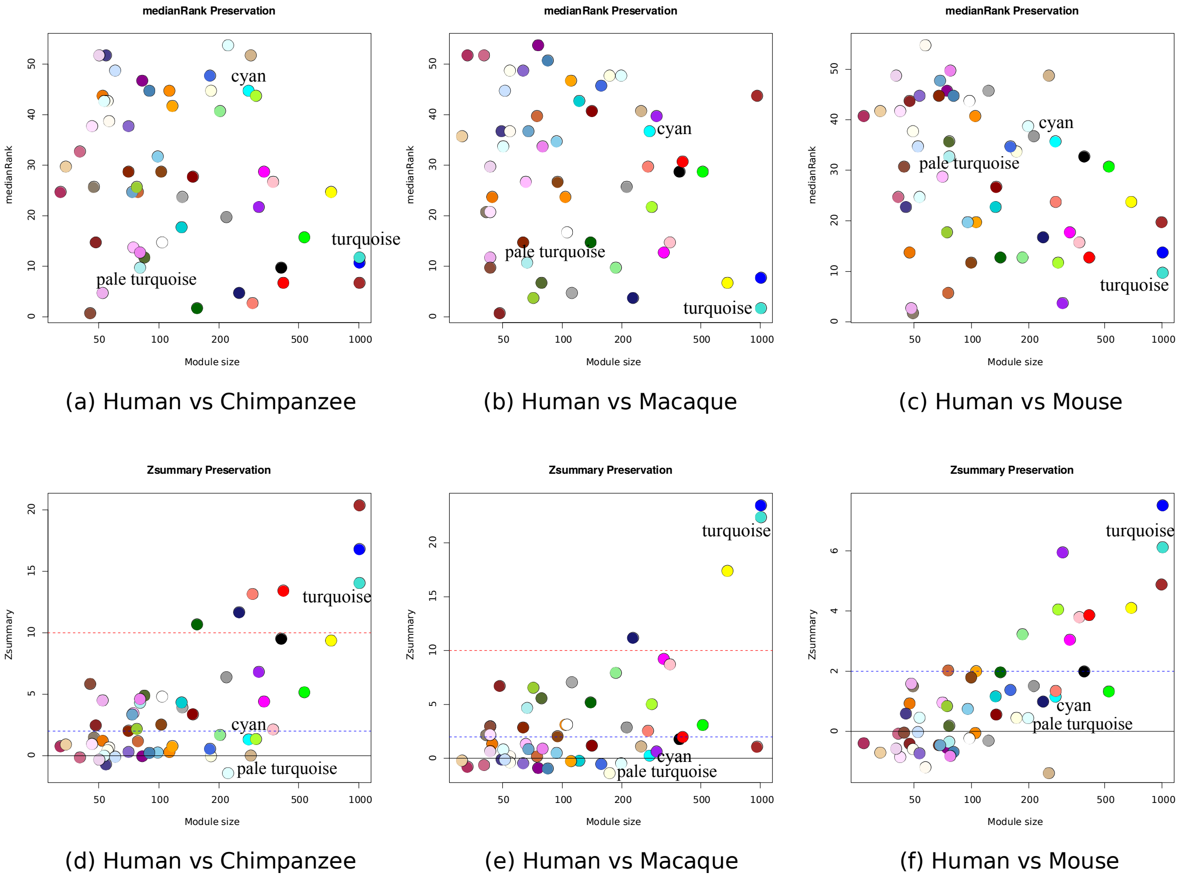

(a) Human vs Chimpanzee

(b) Human vs Macaque

(c) Human vs Mouse

(d) Human vs Chimpanzee

(e) Human vs Macaque

(f) Human vs Mouse

Figure S2: Module preservation statistics comparing signed gene co-expression networks constructed using prefrontal cortex samples from human vs chimpanzee, macaque, and mouse. From both Zsummary (top left) and medianRank (top right) preservation scores using mouse as the reference network, the most preservation is observed for the yellow and brown modules. A Zsummary score greater than 2, but less than 10 indicates moderate preservation, while a score greater than 10 indicates strong module preservation. A low score for medianRank indicates high module preservation. Note that WGCNA includes labels for each module colour by default; however, a selection of modules were highlighted (cyan, turquoise, and pale turquoise) in order to show the modules that contained enriched gene sets related to metabolism.

Table S1: Lists of genes present in the biclusters with the smallest cosine distances highlighted in Figure 8. Genes are coloured based on whether they occur in the brain gene sets (blue), heart gene sets (red), or overlap with both (orange).

	Bicluster[1]	Gene Symbols
Brain vs Brain	0	BAD, PSMB1, PSMA2, MAPK10, NDUFC1, NDUFS8, PSMD9, GAPDH, PSMD14, NDUFS7, RTN4, HTRA2, BID, PARK7,<br>MFN2, SDHB, NDUFB3, NDUFA8, ATG101, NDUFA1, NDUFS1, PSMB2, BECN1, MAP2K2, SEM1, NDUFA5, PSMA1, NDUFAB1<br>NDUFA2, RTN3, PSMA4, EIF2S1, NDUFB5, TXN, PSMB7, NDUFB10, APP, PSMB6, PSMA5, SDHC, CALM2, RPS27A,<br>NDUFS6, NDUFB11, NDUFB9, NRBF2, CAMK2G, NDUFC2, ADAM17, GNAQ, PINK1, PSMD4, PSMB4, NDUFV3, NDUFB4, PSMD6,<br>NDUFS4, NDUFB6, PSMC3, NDUFB8, NDUFS5, SLC25A6, UBB, NDUFA3, CYCS, PSMD1, HRAS, NDUFA11, LRP6, SLC25A5<br>NDUFV2, NDUFB1, NDUFA12, NDUFA6, PSMD13, UBE2L3, NDUFA13, NDUFA4, PSMD12, SDHD, VDAC1, NDUFS3, UBA52, NDUFA7,<br>PSMB3, PSMC5, NDUFB2, PSMD8, NDUFB7, TXN2, PSMA3, PSMB5, PSMA6, PSMA7, MAPK3, PSMD7<br>ITGA10, ATP1B1, TPM3, PRKAG1, ACTG1, ACTB<br>UQCRC1, COX6A1, COX7A2, COX7A2L, COX6B1, COX7C, UQCR11, COX4I1, COX7B, COX5B, UQCRB, COX6B2, COX7A1, UQCRQ,<br>COX6C, UQCRFS1, UQCRH, COX8A, COX5A, UQCR10
	7	WNT16, NOX1, KLC3, AKT2, PIK3R2, WNT2, DVL1, DKK1, WNT3, PSMD11, NFKB1, PSMC4, WNT5B, FZD10,<br>CSNK1A1, WNT5A, IL1A, WNT6, PIK3R3, ATF6, IDE, DUSP1, PPP3CC, PIK3CA, TUBA1B, PLCG1, WNT1, ATG14,<br>TRAF2, RB1CC1, ADORA2A, ATF4, CALML4, APOE, ADRM1, SLC18A1, RAF1, CASP9, CHRM3, IRS4, WNT2B, APC,<br>WNT10A, HSPA5, TUBB2A, ADAM10, APH1B, GNAL, AKT1, SLC6A3, EIF2AK2, WNT9A, PIK3R1, DRD2, PLCB3, UBC,<br>TUBA3E, UCHL1, WNT7A, DKK2, FZD7, KIF5A, DVL2, WNT9B, NAE1, GNAI3, CALM3, UBE2J2, PSMC2, DVL3,<br>WNT4, CAPN2, CASP3, CDK5, FZD6, SLC18A2, CTNNB1, FADD, KIF5C, CHRM1, AXIN2, GRM5, MAP2K1, WNT10B,<br>MAOB, INSR, MFN1, PPID, BCL2L1, PIK3CD, EIF2AK3, RELA, ADCY5, DDIT3, ATG13, CHRNA7, LPL, FZD8,<br>WIPI1, CALML3, CALML5, TUBAL3, ERN1, TH, CSNK2A2, PLCB1, CAMK2A, UBE2G2, DRD1, CHRM5, PRKN, IRS2,<br>WNT7B, TUBB4B, FZD9, LRRK2, MAOA, TUBB, PIK3R4, MME, TUBA3C, UBE2J1, RYR3, DAXX, AGER, CASP12,<br>CSNK2B, CHUK, CSNK1E, PPP3R1, INS, TUBB8, GRIN2B, MAP2K7, ARAF, PIK3C3, WNT11, GNAS, NOS1, MAPK1,<br>XBP1, NOS2, PSMC6, TUBB1, AXIN1, IKBKB<br>PRKAG2, SGCA, CACNG1, MYL2, PRKAB1, TNNC1, ITGB6, ITGA4, PRKAG3, IGF1, TNNT2, CASQ2,<br>ATP1A2, TNNI3, CACNG6, PRKAB2, PRKAA1, ATP1A4, MYBPC3, ITGA11, CACNG8, ITGA9, TTN, CACNA2D3<br>ACTC1, ACE, SLC9A7, TPM4, CACNB1, DES, SLC8A1, LAMA2, MYH6, MYL4, TPM2, SLC9A6,<br>DMD, CACNG5, CACNG4, ITGA8, CACNG3, MYH7, TGFB2, SLC8A3, LAMA1, ATP1B4, EMD<br>COX6A2, ATP2A1, ATP2A3, TNF, CACNA1S
	3	BAD, WNT16, NOX1, DKK4, TUBB4A, KLC3, AKT2, GRIN2D, PIK3R2, WNT2, MAPK8, DKK1,<br>WNT3, NFKB1, MAPK10, ATG2A, AMBRA1, WNT5B, FZD10, CSNK1A1, WNT5A, CAPN1, IL1A, APC2,<br>RTN4, HTRA2, BID, WNT6, NFE2L2, APH1A, PIK3R3, ATF6, DUSP1, PIK3CA, TUBA1B, GPR83,<br>WNT1, IL1B, ATG14, GNAI1, ATF4, SLC18A1, CASP9, CHRM3, IRS4, KRAS, WNT10A, TXN,<br>KLC4, TUBB2A, TUBB2B, PLCB2, ADAM10, PPP3CA, GNAL, TP53, SLC6A3, PSEN2, WNT9A, RPS27A,<br>SNCA, NRBF2, CAMK2G, DRD2, UBC, ITPR1, WNT8A, ADAM17, UCHL1, WNT7A, DKK2, FZD7,<br>KIF5A, MCU, FZD1, BRAF, WIPI2, WNT9B, GNAI3, UBE2J2, DVL3, GRIN2C, LRP5, WNT4,<br>FZD5, CASP3, CDK5, FZD6, CYBB, ATG2B, SLC18A2, FRAT1, TNFRSF1A, TUBA1A, TUBA1C, CTNNB1,<br>FADD, KIF5C, CHRM1, GRM5, GPR37, INSR, BCL2L1, PIK3CD, EIF2AK3, RELA, HRAS, ATG13,<br>CHRNA7, TUBB6, CDK5R1, GRIN1, ULK1, CALML3, CALML5, TUBAL3, ERN1, TH, FZD2, FRAT2,<br>CSNK2A2, GRIN2A, DRD1, CHRM5, IRS2, BACE1, WNT7B, FZD9, TUBB, MME, TUBA3C, UBE2J1,<br>DAXX, CASP12, NRAS, PPP3R1, UBA52, INS, TUBB3, TUBB8, GRIN2B, MAP2K7, PSEN1, WNT11,<br>NOS1, XBP1, NOS2, TUBB1, PLCB4, AXIN1, FZD3<br>CACNG7, ITGB8, PRKAG2, CACNG1, ITGA4, PRKAG3, SLC8A2, CACNG6, PRKAA1, ATP1A4, AGT, ITGA11,<br>ITGB7, CACNG8, TPM3, CACNA2D4, LMNA, ITGA5, ITGA2, CACNG2, TPM4, ATP1B3, ACTG1, LAMA2,<br>MYH6, MYL4, ITGA1, ITGB3, CACNG5, CACNG4, ITGA3, ACTB, ITGA8, EDN1, ITGA2B, SLC9A1,<br>CACNG3, ITGA6, TGFB2, SLC8A3, CACNA2D2, ATP1B4, EMD<br>COX6A1, COX4I2, IL6, CACNA1D, COX6B2, ATP2A1, TNF, CACNA1S, CACNA1F
Heart vs Heart	8	PSMB1, PSMA2, DVL1, PPIF, PSMD3, PSMD11, NDUFC1, NDUFS8, PSMD9, GAPDH, NDUFS7, PARK7,<br>MFN2, AKT3, SDHB, NDUFB3, NDUFA8, APAF1, ITPR2, LRP1, NDUFA1, NDUFS1, KLC1, TRAP1,<br>MAP2K2, TUBA4A, SEM1, CALML4, NDUFAB1, FAS, APOE, NDUFA10, ADRM1, NDUFA2, UBE2G1, PSMA4,<br>WNT2B, NDUFB5, PSMB7, APH1B, NDUFA9, NDUFB10, AKT1, PSMB6, SDHC, EIF2AK2, NDUFS6, CAMK2B,<br>NDUFB11, NDUFB9, NDUFC2, SLC25A4, TUBA3E, CASP8, GNAQ, SNCAIP, PINK1, NDUFS2, PSMD4, PSMB4,<br>CALM3, NDUFV3, NDUFB4, PSMD6, NDUFS4, NDUFB6, VDAC2, PSMC3, NDUFB8, APBB1, NDUFV1, NDUFS5,<br>WNT10B, UBB, MAOB, NDUFA3, ADCY5, NDUFA11, KLC2, PSMD2, LRP6, LPL, FZD8, NDUFV2,<br>BACE2, PLCB1, NDUFB1, TUBA8, CAMK2A, NDUFA12, UBE2G2, NDUFA6, PSMD13, PRKACA, UBE2L3, NDUFA13,<br>MAPT, TUBB4B, LRRK2, HSD17B10, NDUFA4, MAP3K5, SDHA, RYR3, PTGS2, SDHD, CSNK2B, PSENEN,<br>VDAC1, NDUFS3, NDUFA7, PSMB3, TUBA3D, ARAF, VDAC3, NOX4, PSMC5, GNAS, NDUFB2, PSMD8,<br>NDUFB7, TXN2, PSMA3, PSMB5, PSMA6, PSMA7<br>TGFB1, ATP1A3, SGCA, MYL2, TNNC1, ITGB6, IGF1, TNNT2, TGFB3, TNNI3, HRC, ITGB4, MYBPC3, TPM1, ITGA10<br>ATP1B1, ITGA9, TTN, ACTC1, MYL3, CACNB2, CACNB1, DES, PRKAG1, CACNB4, TRDN, TPM2, RYR2, ITGB5, MYH7,<br>LAMA1, SGCG<br>UQCRC1, COX7A2, COX7A2L, COX6B1, COX7C, UQCR11, COX4I1, COX7B, COX5B, UQCRC2, CACNA1C, UQCRB, COX6A2, COX7A1,<br>UQCRQ, COX6C, UQCRFS1, UQCRH, COX8A, COX5A, CYC1, UQCR10, UQCRHL
Brain vs Heart	8	PSMB1, PSMA2, DVL1, PPIF, PSMD3, PSMD11, NDUFC1, NDUFS8, PSMD9, PSMC4, GAPDH, PSMD14,<br>NDUFS7, RTN4, BID, NFE2L2, PARK7, MFN2, AKT3, SDHB, NDUFB3, NDUFA8, APAF1, ITPR2,<br>LRP1, NDUFA1, NDUFS1, PSMB2, KLC1, TRAP1, MAP2K2, TUBA4A, SEM1, NDUFA5, CALML4, PSMA1,<br>NDUFAB1, FAS, APOE, NDUFA10, ADRM1, UBA1, NDUFA2, RAF1, UBE2G1, PSMA4, WNT2B, NDUFB5,<br>PSMB7, APH1B, NDUFA9, NDUFB10, GNAL, AKT1, PSMB6, PSMA5, SDHC, EIF2AK2, CALM2, NDUFS6,<br>CAMK2B, NDUFB11, NDUFB9, NDUFC2, SLC25A4, TUBA3E, CASP8, GNAQ, PINK1, NDUFS2, PSMD4, PSMB4,<br>, NAE1, CALM3, NDUFV3, PSMC2, NDUFB4, PSMD6, NDUFS4, NDUFB6, VDAC2, PSMC3, NDUFB8, APBB1,<br>NDUFV1, NDUFS5, WNT10B, UBB, MAOB, NDUFA3, INSR, PPID, CYCS, ADCY5, NDUFA11, KLC2,<br>PSMD2, LRP6, LPL, FZD8, SLC25A5, NDUFV2, PLCB1, NDUFB1, TUBA8, CAMK2A, NDUFA12, UBE2G2,<br>NDUFA6, PSMD13, PRKACA, UBE2L3, NDUFA13, MAPT, TUBB4B, LRRK2, HSD17B10, NDUFA4, MAP3K5, SDHA,<br>RYR3, PTGS2, SDHD, CSNK2B, PSENEN, VDAC1, NDUFS3, NDUFA7, PSMB3, TUBA3D, ARAF, VDAC3,<br>NOX4, PSMC5, GNAS, NDUFB2, PSMD8, NDUFB7, TXN2, PSMC6, PSMA3, PSMB5, PSMA6, PSMA7,<br>PSMD7<br>TGFB1, ATP1A3, SGCA, MYL2, TNNC1, ITGB6, IGF1, TNNT2, CASQ2, TGFB3, TNNI3, HRC, ITGB4, MYBPC3, ITGA7,<br>TPM1, ITGA10, ATP1B1, ITGA9, TTN, MYL3, ITGA2, CACNB2, CACNB1, DES, PRKAG1, CACNB4, TRDN, TPM2, RYR2,<br>ITGB5, MYH7, LAMA1, EMD, SGCG<br>UQCRC1, COX7A2, COX7A2L, COX6B1, COX7C, UQCR11, COX4I1, COX7B, COX5B, UQCRC2, UQCRB, COX6A2,<br>COX7A1, UQCRQ, COX6C, UQCRFS1, UQCRH, COX8A, COX5A, CYC1, UQCR10, UQCRHL

[1]Bicluster number can be interpreted using the following image indicating bicluster location:

0	3	6
1	4	7
2	5	8
